# Quality assessment in surgery: mission impossible?

**DOI:** 10.1186/1754-9493-4-18

**Published:** 2010-11-21

**Authors:** Daniel Dindo, Pierre-Alain Clavien

**Affiliations:** 1 University Hospital Zurich, Department of Surgery, Raemistr. 100, CH-8091 Zurich, Switzerland

## Quality in Surgery

Safety and quality have become prominent criteria by which surgical care is evaluated. Physicians and hospitals are increasingly asked for evidence addressing these areas. Such demands arise from better educated patients and more demanding payers. Patients are starting to use such documentation to select their practitioners and the site for their care [1]. In addition, payers are seeking to use such data to direct selected patient population to particular providers. Therefore, health service policy makers are seeking to develop and implement quality indicators that can be appropriately applied to medical practice. However, up to now, such endeavours have failed. There is still a dramatic shortage of quality assessment programs in surgery. In the United States, large databases such as the National Surgical Quality Improvement Program (NSQIP) were established in 1991 in Veteran Affairs Hospitals, used to record surgical outcome. However, only a minority of US hospitals has joined this program. Today, there is still no general concept on how to assess surgical quality for most of the hospitals neither in the United States nor outside.

Quality assessment in surgery requires different components in order to be valuable. First of all, we need to talk the same language. There is still no common sense on how to report surgical outcome. In 2004, we proposed a classification system for surgical complications, which has gained much acceptance in the surgical community with over 400 citations [2,3]. It certain regions, e.g. in Sweden, the registration of complications according to this classification system has even becomes mandatory in upper-GI surgery.). Another key task in assessing the quality of surgery is to take into account the degree of risk of the patients studied. For example, we may not expect the same results comparing in an elderly obese patient operated urgently for acute cholecystitis vs elective cholecuytectomy in a young patient. Whenever we speak of surgical 'quality', the case-mix must be considered. This last factor is most often poorly reported in the current literature due to the lack of an accepted strategy to adjust for the patients' risk. This severely hampered interpretation of quality reports. Another important issue is the reliability of the published data. Without truthful data, quality assessment is at best misleading. This subject is hardly explored in surgical literature. In a prospective study, we have demonstrated that outcome data that is collected by residents is not reliable [4]. Data collection using dedicated personnel such as in the NSQIP would be advisable but only few centers come with data managers or study nurses. And last but not least, an important factor of quality assessment is a common definition of the term 'quality' itself. In 1980, Donabedian defined quality care as 'that kind of care which is expected to maximize an inclusive measure of patient welfare, after one has taken account of the balance of expected gains and losses that attend the process of care in all its parts [5]. This definition points to another problem: Quality has to be seen from different angles - not only the patient has its demands on the health care system but also governments and insurances. Therefore, the definition of quality may widely differ between patients, the society, the administrators, and the health care policy makers. Taken as a whole, quality assessment remains a challenging task, as we still lack standardized or widely accepted tools to convincingly perform such quality assessment in many areas. As long as those inconsistencies are not solved, quality assessment will be more a mythical creature than a powerful tool to improve quality at reasonable costs.

## In the Land of the Rising Sun

**Figure 1:**
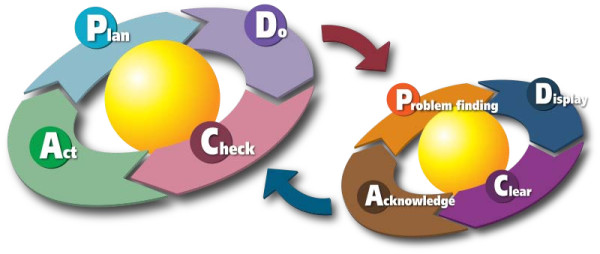
**The PDCA cycle**.

What we could not attain in surgery, so far, is already reality in private industry. Since decades, quality assurance programs are well-established in manufacturing and trading. The effort to improve quality in production processes was first made in Japan in the early 50ties, importantly contributing to the economic success of this country. One of the most important ingredients of Japanese firms' high performance is the fact that all employees are all involved in quality improvement activities. These activities have been summarized under the term 'Kaizen'. Kaizen, which in Japanese means good (zen) change (kai), is a philosophy that motivates people to constantly improve their surroundings. The Kaizen principles were first introduced by Toyota as part of their Total Quality Management (TCM), thus leading Toyota to one of the companies with the highest quality products. An important principle of Kaizen is to work according to the PDCA circle (**P**lan-**D**o-**C**heck-**A**ct) (Figure 1), which enables a constant improvement of the environment. This circle (also called Deming's circle) is deemed to ease the implementation of new processes. The strength of Deming's concept lies in its apparent simplicity. The concept of feedback mechanism is today firmly established in industry (especially in Japan) as well as in science. In surgery, this concept seems not to be fully adopted. While apparently easy to understand, it is often difficult to accomplish on an on-going basis, and changes are often introduced very slowly into surgical practice. For example, health policy makers are recommending since more than a decade that certain high-risk oncologic procedures should be concentrated into high-volume centers. Several studies have shown dramatic differences regarding surgical and oncological outcome between low- and high volume centers [6,7]. However, significant outcome gaps between high- and low-volume centers still persist. Hence, data of strong evidence are often just ignored fro the sake of personal or local interests. The surgical cycles is more of a 'PDC' cycle (Plan-Do-Check) often missing the most important 'A' (Act). If we followed the PDCA cycle more consequently, a big step towards good quality would have been made.

## Where do we stand?

In recent years, a plethora of papers have been published on quality assessment in surgery. But, did the way of quality assessment and the quality of care itself improve? There is still no consensus on how to adjust for the case mix and on how to define surgical outcome and complications, respectively. We still lack of benchmarks in many surgical fields hampering the reading of surgical outcome data; striking evidence such as the outcome-volume relation is just disregarded remaining without medical or political consequences in many areas and places; reliability of self-reported data might be poor and professional data collectors might not be afforded. - So, let's be honest - we did not come far in the last decades. Quality assessment in surgery is still a neglected subject, and very few seem really to care. We would contend that it is mandatory for all surgeons to understand and properly apply quality tools, so that we can police our own practices before others, such as insurance companies and hospital administrators, will do it for us. We have to make that mission possible.
